# Early impairment of myocardial deformation assessed by regional speckle-tracking echocardiography in the indeterminate form of Chagas disease without fibrosis detected by cardiac magnetic resonance

**DOI:** 10.1371/journal.pntd.0008795

**Published:** 2020-11-30

**Authors:** Minna Moreira Dias Romano, Henrique Turin Moreira, José Antônio Marin-Neto, Priscila Elias Baccelli, Fawaz Alenezi, Igor Klem, Benedito Carlos Maciel, Joseph Kisslo, André Schmidt, Eric J. Velazquez

**Affiliations:** 1 Cardiology Center of the Medical School of Ribeirão Preto, Internal Medicine Department, University of São Paulo (USP), Brazil; 2 Cardiology, Department of Medicine, Duke University School of Medicine, Durham, NC, United States of America; University of South Carolina, UNITED STATES

## Abstract

Chagas disease (CD) will account for 200,000 cardiovascular deaths worldwide over the next 5 years. Early detection of chronic Chagas cardiomyopathy (CCC) is a challenge. We aimed to test if speckle-tracking echocardiography (STE) can detect incipient myocardial damage in CD. METHODS: Among 325 individuals with positive serological tests, 25 (age 55±12yrs) were selected to compose the group with indeterminate form of Chagas disease (IFCD), based on stringent criteria of being asymptomatic and with normal EKG/X-ray studies. This group was compared with a group of 20 patients with CCC (55±11yrs) and a group of 20 non-infected matched control (NC) subjects (48±10yrs). CD patients and NC were submitted to STE and CD patients were submitted to cardiac magnetic resonance (CMR) with late gadolinium administration to detect cardiac fibrosis by the late enhancement technique. Global longitudinal strain (GLS), circumferential (GCS) and radial strain (GRS) were defined as the average of segments measured from three apical view (GLS) and short axis views (GRS and GCS). Regional left ventricular (LV) longitudinal strain (Reg LS) was measured from each of the 17 segments. Twist was measured as systolic peak difference between basal and apical rotation and indexed to LV length to express torsion. RESULTS: STE global indices (GLS, GCS, twist and torsion) were reduced in CCC vs NC (GLS: -14±6.3% vs -19.3±1.6%, p = 0.001; GCS: -13.6±5.2% vs -17.3 ±2.8%; p = 0.008; twist: 8±7° vs 14±7°, p = 0.01 and torsion: 0.96±1°/cm vs 1.9±1°/cm, p = 0.005), but showed no differences in IFCD vs NC. RegLS was reduced in IFCD vs NC in four LV segments: basal-inferior (-16.3±3.3% vs -18.6±2.2%, p = 0.013), basal inferoseptal (-13.1±3.4 vs -15.2±2.7, p = 0.019), mid-inferoseptal (-17.7±3.2 vs -19.4±2, p = 0.032) and mid-inferolateral (-15.2±3.5 vs -17.8±2.8, p = 0.014). These abnormalities in RegLS occurred in the absence of myocardial fibrosis detectable with CMR in nearly 92% of subjects with IFCD, while myocardial fibrosis was present in 65% with CCC. CONCLUSION: RegLS detects early regional impairment of myocardial strain that is independent from fibrosis in IFCD subjects.

## Author summary

Regional longitudinal strain is reduced in IFCD before significant fibrosis. As CD progresses to CF, global STE measurements of left ventricle (GLS, GCS, twist and torsion) and GLS of right ventricle are reduced.

Early abnormal strain pattern of CD comprises inferior, septoinferior and inferolateral segments of LV. Recognizing the regional strain pattern of Chagas cardiomyopathy may help identifying the disease even at early stages.

## Introduction

More than one century after its discovery in 1909 [1] Chagas disease (CD), caused by infection with the *Trypanosoma cruzi* protozoan, is still a major public health problem in Latin America [2] and, due to migratory moves during the last decades, also in non-endemic areas, such as the United States and some European countries [3–5].

An estimated 50–60% of subjects chronically infected with the *T*. *cruzi* remain throughout life with the indeterminate form of CD (IFCD) i.e. they do not show any clinical signs of organic involvement. Although the concept of the IFCD has been occasionally challenged [6], its definition—as applied to the condition of an infected individual who is asymptomatic and without abnormalities in the physical exam, the 12-lead electrocardiography (EKG) and the radiological examination of the heart, esophagus and colon—is time honored and maintained in recent guidelines [7, 8]. This is mainly due to the belief that as long as the infected individual remains with this indeterminate form of the disease, the prognosis is good, and the risk of death is similar to that of non-infected matched control (NC) subjects. In contrast, chronic Chagas cardiomyopathy (CCC) is the most serious of the various clinical manifestations of CD, responsible for a high morbidity and mortality burden that includes a most common cause of sudden death and up to 11.0% of cases of heart failure in endemic regions [2, 9–11].

Two main issues regarding the IFCD remain unsolved. The first is related to which genetic or acquired factors determine, in a specific patient, the installation of the cardiomyopathy [12, 13]. The second is whether any anatomical or functional abnormality detected in individuals with the IFCD would herald the subsequent development of CCC in the natural history of the disease [14].

The second issue is particularly relevant when it is known that no etiological treatment has been proven to benefit patients already with the CCC [15], whereas early treatment of individuals with the IFCD may be a more promising approach [14]. Also, a vaccine solution could be in the future available for use in humans, on the basis of promising results with the murine experimental model of chronic *T*. *cruzi* infection, showing reduced parasite load and cardiac damage [16]. In this context, diagnostic approaches capable of predicting the appearance of CCC and identifying early signs of myocardial damage would be most useful, especially if they are shown to have prognostic meaning in the natural history of CD.

Two-dimensional speckle-tracking echocardiography (STE), a method to quantitate global and regional myocardial deformation [17, 18] has been shown to detect early myocardial deformation alterations in some non-ischemic cardiomyopathies [19–21].

Preliminary studies in IFCD individuals using STE described mixed results, failing to show reduction of LV global strain but reported lower values of radial strain in the mid-inferior segment in individuals with the IFCD as compared with NC subjects [22]. In another study, lower longitudinal velocities, but not strain, were described in a small group of 8 individuals without abnormalities in the 12-lead EKG and with normal LV at echocardiography [23]. In addition, in a subgroup of asymptomatic patients with Chagas’ disease, regional LV longitudinal strain (RegLS) was found to be reduced compared with healthy individuals in two LV segments [24]. However, these findings were not confirmed by another study of patients with CD but with no evidence of cardiac involvement, in which global and segmental LV strain was similar to that of NC subjects; in that study, lower RegLS, global longitudinal, radial and circumferential LV strain appeared only in a subgroup of CD patients who have myocardial fibrosis detected with cardiac magnetic resonance (CMR) imaging [25].

We hypothesized that RegLS or global STE can identify early myocardial dysfunction in IFCD. Thus, the aims of this study were (1) to compare LV systolic global (longitudinal (GLS), radial (GRS) or circumferential (GCS)) and RegLS values between individuals with IFCD, age matched NC and patients with the CCC; and (2) to observe if LV strain abnormality correlates with presence and the extent of myocardial fibrosis measured by cardiac magnetic resonance imaging (CMR).

## Methods

### Study design

This cross-sectional study was designed to compare ventricular strain values between Chagas subjects (IFCD and CCC) and an age matched NC individuals. Sample size calculation was based on a presumed difference of 15% on LV global longitudinal strain values among the three groups and resulted in an estimation of 23 individuals in each study group [26].

### Study population

To compose the IFCD group, individuals with positive serology for CD and no symptoms of cardiac disease, normal EKG and normal chest X-ray were recruited searching hospital medical records during the years 2015–2016. Inclusion criteria were: two positive serology tests for CD, age ≥18 years old. Exclusion criteria were: age ≥ 85 years old; presence of any other cardiovascular disease, motor disability limiting cardiac tests; arterial hypertension; significant psychiatric diseases, renal failure or hepatic cirrhosis. Patients who already had the diagnosis of CCC were enrolled consecutively from the outpatient clinics of the University Hospital of the Medical School of Ribeirão Preto, University of São Paulo Brazil. Healthy volunteers who were age and gender matched to the IFCD individuals were invited to participate and had serological tests for CD that yielded negative results in all cases. All recruited subjects underwent standard 12-lead EKG, upright chest X-rays and rest transthoracic echocardiography. Subjects with CD underwent CMR at the same day of echocardiography. NC volunteers were submitted to the same exams except for CMR. All EKG abnormalities such as complete right bundle branch block (RBBB), complete left bundle branch block (LBBB) and left anterior fascicular block (LAFB) were defined following previous published guidelines (20). Any kind of atrio-ventricular block was classified simply as an "AV block".

The institutional research ethics committee approved the study protocol (process number HCRP **17096/2015**), which was conducted in accordance with the Helsinki Declaration. Written informed consent was obtained from all participants.

### Echocardiography

All transthoracic echocardiography images were collected prospectively to guarantee the best image quality, by the same echo certified examiner (PB), who was trained to acquire complete conventional echocardiography and image sequences dedicated to speckle tracking analysis including left ventricular (LV) twist and torsion. Images were acquired with Vivid E9 or S6 (GE Healthcare, Horten, Norway), with a phased-array transducer of 1.4–4.6 MHz. Image acquisition strictly followed previous published guidelines [27, 28]. Briefly, LV apical images were acquired at the longest possible LV axis, thus avoiding shortening of LV; LV short axis images were acquired at basal, papillary muscle and apex levels. Apex level was defined as the most apical possible short axis, based on the apical four-chamber view. Images were recorded with EKG tracings in at least 3 consecutively cardiac cycles in quiet respiration. All images were acquired at a frame-rate of 55–90 frames/sec.

Images were archived as GE dedicated raw data and analyzed by another researcher (MMDR), blinded to the subject study group, with EchoPac software (GE Vingmed Ultrasound AS) version 112.

Conventional echocardiographic left (LV) and right (RV) ventricular dimensions and function were collected as follows: LV linear transversal dimension at diastole and systole (LVDd, LVDs), longitudinal dimension measured as an average of longitudinal linear dimensions in two-chamber view and four-chamber views, end-diastolic volume (LV EDV), end-systolic volume (LV ESV) and ejection fraction (LVEF) derived from Simpson’s modified rule [29]; RV linear transversal dimensions at basal level of apical four-chamber view, and Doppler tricuspid annular systolic velocity (S’). Left ventricle wall motion score was calculated based on a 17 segmentation mode as previously described [30].

### Two-dimensional echocardiography strain analysis

A single experienced physician (MMDR) performed all two-dimensional strain analyses using the three LV apical views, a 4-chamber dedicated RV view and short LV axis view at basal, mid papillary and apical levels. The second of the three acquired cardiac cycles was chosen for analysis whenever possible. All strain measurements were collected as "full thickness” (or meso) myocardium and end-systolic strain values (ESS) were measured, avoiding post-systolic strain measurements. Reference time point was manually set to the beginning of the QRS. End-systole [31] was defined at the time of aortic closure from Doppler velocity signal at LV outflow tract when measuring LV strain and at the time of pulmonary valve closure from Doppler signal of RV outflow tract when measuring RV strain. Endocardial border was traced at end-systole and the region of interest adjusted to exclude the pericardium. Integrity of tracking was checked as well as integrity of strain curves and measured peaks. Segments with persistently inadequate tracking were excluded from analysis. A maximum of 2 excluded segments was deemed tolerable for LV and of 1 segment for RV 4-chamber strain analysis.

Longitudinal strain (LV and RV) was measured as end-systolic strain (ESS) from full thickness myocardial, which is named mid-layer in GE EchoPac software as a default. Global longitudinal strain (GLS) was measured automatically by the system as an average of 17 segmental strain values from three projections of LV in apical view. LV segmental strain from each of 17 LV segments was similarly collected. Circumferential and radial strain were also measured as ESS. Global circumferential (GCS) and radial strain (GRS) were calculated as an average of each segmental value from a 18- segmentation model, derived from short axis views at basal (6 segments), mid (6 segments) and apical (6 segments) LV levels. All strain values were expressed as % changes. LV twist value was collected from the derived curve of apex-basal rotation. The end-systolic peak of the derived subtraction curve was manually measured using the aortic valve closure time mark as the time reference point into the software. Twist value was expressed in degrees and was divided by LV longitudinal dimension to construct the torsion value, the former being expressed in degrees/cm [32].

### Cardiac magnetic resonance

Cardiac magnetic resonance images were acquired from all Chagas subjects (IFCD and CCC groups) the same day as the echocardiography recording. Cine loops were acquired using a steady-state free precession pulse sequence gated by EKG, obtained with a 1.5 Tesla scanner Achieva (Philips, Netherlands), with a predefined protocol as follows: repetition time of 3.8 msec, echo time of 6 msec, flip angle of 45°, 30 acquisition phases, 256x160 matrix and 360–400 mm field of view. LV and RV were analysed in short axis slices (thickness of 8 mm, 2mm gap between slices). For myocardial tissue evaluation and fibrosis detection, subjects received 0.2 mmol/Kg gadolinium-based contrast (Ominiscan, GE Healthcare) by intravenous infusion. T1-weighted inversion recovery fast gradient echo pulse sequence was applied 10 min after the contrast injection. LV slices with 10mm of thickness and no gap were accessed to identify gadolinium enhancement. Exams were recorded in the DICOM pattern in HCRP-Brazil. Images were transferred to a dedicating reading software (Duke precession Saas) following HIPPA privacy guidelines and de-identified for blind analysis. Two experienced physicians were responsible for reading CMR images (IK, SD), blinded to both echocardiography data and subject group. Simpson’s disk summation technique was used to determine ventricular volumes and ejection fraction from short axis slices. RV outflow tract was not included into RV volumes. Endocardial borders were manually delineated excluding papillary muscles and trabeculae. To detect myocardial scar, hyper-enhancement signal was visually scored by using a 17-segmentation model with a five point scale for each segment, in which 0 = no hyper-enhancement, 1 = 1–25%, 2 = 26–50%, 3 = 51–75% and 4 = 76–100%. We calculated total size of LV scarring (% of myocardium) by summing the regional scores and dividing by the total numbers of segments analyzed (n = 17). The percentages of any amount of scar (> 0%) in any wall (%LV) for both IFCD and CCC Chagas groups were recorded.

### Reproducibility

Echocardiography strain parameters GLS, GCS, GRS and RegLS were tested for reproducibility in a subset of 15 subjects randomly selected proportionally to the three studied groups (IFCD, CCC and NC). For intra-observer variability, a re-reading (MMDR) was performed at least one month after the first reading, blinded to the previous one. For inter-observer variability a second experienced echocardiographer physician (FA) performed analyses using the same technique previously described, also blinded to both first reader analysis and individual study group. Comparisons of results were done with intra-class correlation coefficient (ICC) to assess absolute agreement and a coefficient of variation (CV) determined by the standard deviation divided by the mean and expressed as percentage.

### Statistical analysis

Sample size calculation was done using standard methods [26]. Data were expressed as mean ± standard deviation. Continuous data were tested for normality distribution with Shapiro-Wilk tests and for equal variances with Barlett’s tests. Data between groups (NC vs IFCD vs CCC) were compared using analysis of variance (ANOVA) followed by Tuckey post-hoc pair-wise analysis. Student’s t tests, Chi-Square tests or Kruskal-Wallis tests were used as appropriate. Correlation between average LV GLS or RV GLS and fibrosis percent was assessed by Pearson’s correlation coefficient. Significance level was set as p <0.05. All statistical analyses were performed using Stata 14.0 (StataCorp, College Station, TX).

## Results

**Fig 1:**
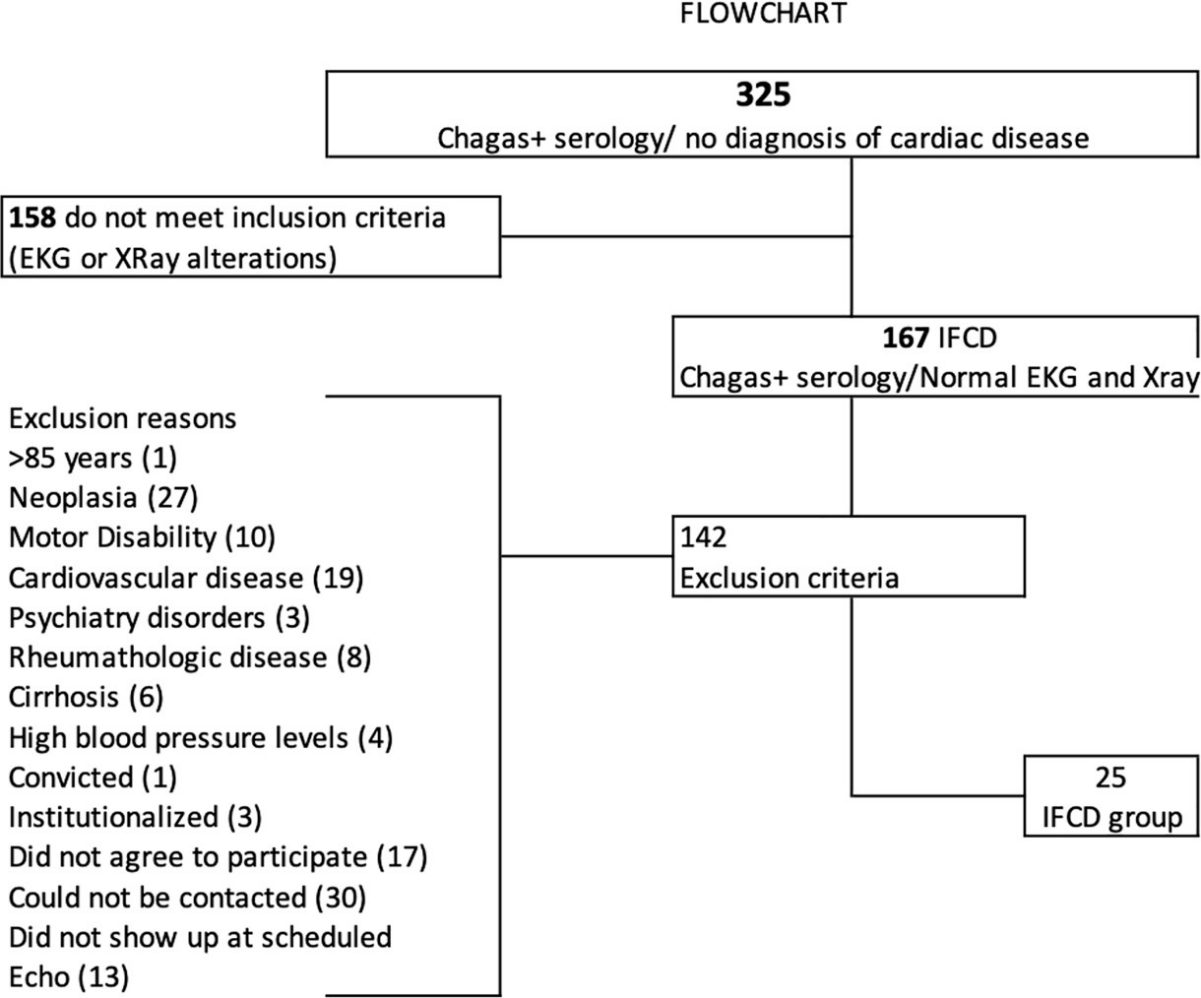
Flowchart of selection of Indeterminate Form Chagas Disease (IFCD) group of individuals with Chagas positive serologies.

Population screening of IFCD individuals is described in flowchart (**Fig 1**). We reviewed the hospital records of all 325 consecutive asymptomatic subjects with positive serological Chagas tests evaluated at our institution from 01 of January of 2015 to 31 of December of 2016. After the exclusion criteria as shown in **Fig 1**, the IFCD group was composed of 25 individuals. Another subset of 20 consecutive screened patients with Chagas disease and clinical signs of heart involvement constituted the CCC group.

Baseline characteristics of the 20 patients with CCC, the 25 with IFCD and the 20 individuals of the normal controls group (NC) are described in **Table 1**. Demographic and most clinical characteristics were comparable among the three groups but NYHA class > 1, EKG abnormalities and cardiomegaly were detected only in the CCC group.

Conventional echocardiographic and deformation parameters analyzed in the 3 groups are shown in **Table 2**. When comparing the NC and the IFCD groups, echocardiography conventional variables did not differ statistically, except for a slightly increased LV end-systolic diameter of 3.0 ±0.5 vs 2.8±0.4 (p = 0.023).

**Table 1:** Demographic and clinical characteristics across the groups.

	NC	IFCD	CCC	p-value
n	20	25	20	
Age (years)	48 ± 10	55 ± 12	53 ± 12	0.120
Male	11 (55%)	15 (60%)	6 (30%)	0.112
Body surface area (m2)	1.78 ± 0.17	1.86 ± 0.27	1.72 ± 0.20	0.105
Heart rate (bpm)	73.9 ± 9.7	70.6 ± 10.2	66.8 ± 12	0.120
Systolic blood pressure (mmHg)	122.0 ± 13.5	129.6 ± 11.1	121.2 ± 18.6	0.100
Diastolic blood pressure (mmHg)	74.5 ± 9.9	75.7 ± 10.1	69.7 ± 8.9	0.112
Diabetes mellitus	0	2 (8%)	1 (5%)	1.000*
Smoking	0	3 (12%)	3 (15%)	1.000*
NYHA				
I	20 (100%)	25 (100%)	12 (60%)	-
II	0	0	6 (30%)	-
III	0	0	2 (10%)	-
EKG abnormalities				-
Atrial fibrillation	0	0	2 (10%)	-
First degree AV block	0	0	3 (15%)	
LBBB	0	0	2 (10%)	-
RBBB	0	0	1 (5%)	-
LAFB	0	0	7 (35%)	-
QRS low voltage	0	0	1 (5%)	
X-ray cardiomegaly	0	0	4 (20%)	-

*Comparisons between IFCD group and the CCC group.

IFCD = Indeterminate Form Chagas disease; CCC = Chronic Chagas Cardiomyopathy; NC = Non-infected control individuals; NYHA = New York Functional Class; EKG = Electrocardiography; AV = atrio ventricular; LBBB = Left bundle branch block; RBBB = Right bundle branch blockç LAFB = Left anterior fascicular block.

**Table 2:** Echocardiographic parameters of LV and RV structure and function across the groups.

	NC	IFCD	CCC	p-value
**Conventional**				
LV end-diastolic dimension (cm)	4.3 ± 0.4	4.5 ± 0.4	5.0 ± 0.9#	0.12
LV end-systolic dimension (cm)	2.8 ± 0.3	3.0 ± 0.5*	3.9 ± 1.1#	0.02
LV end-diastolic volume (mL)	72 ± 14	79 ± 23	116 ± 65#	0.35
LV end-systolic volume (mL)	29 ± 8	31 ± 12	67 ± 58#	0.69
LV ejection fraction (%)	60 ± 5	61 ± 6	47 ± 14#	0.67
RV basal end-diastolic diameter (cm)	3.4 ± 0.5	3.4 ± 0.6	3.5 ± 0.6	0.86
RVS' (cm/s)	13 ± 2	13 ± 2	11 ± 2#	0.64
Wall motion score index	1.0±0	1.01±0.04	1.48±0.51#	0.69
**Speckle Tracking-derived**				
LV global longitudinal strain (%)	-19.3 ± 1.6	-18.8 ± 2.8	-14.0 ± 6.3 #	0.50
LV global circumferential strain (%)	-17.3 ± 2.8	-15.6 ± 3.3	-13.6 ± 5.2#	0.07
LV global radial strain (%)	34.2 ± 10.3	28.4 ± 14.6*	25.8 ± 15.4	0.04
LV twist (degree)	14 ± 7	11 ± 11	8 ± 7 #	0.22
LV torsion (degree/mm)	1.9 ± 1.0	1.3 ± 1.4	0.96 ± 1.0 #	0.10
RV free wall longitudinal strain (%)	-26.4 + 6.4	-24.5 ± 4.9	-20.2 ± 1.1#	0.30
RV free wall and septum strain (%)	-20.9 ± 4.7	-19.9 ± 4.8	-16.4 ± 3.1#	0.49

* p <0.05 NC vs IF

# p <0.05 NC vs CF

IFCD = Indeterminate Form Chagas Disease

CCC = Chronic Chagas Cardiomyopathy

LV = left ventricle; RV = right ventricle; RVS’ = S wave from tissue Doppler of tricuspid valve; IFCD = Indeterminate Form Chagas disease; CCC = Chronic Chagas Cardiomyopathy; NC = Non-infected control individuals.

**Fig 2:**
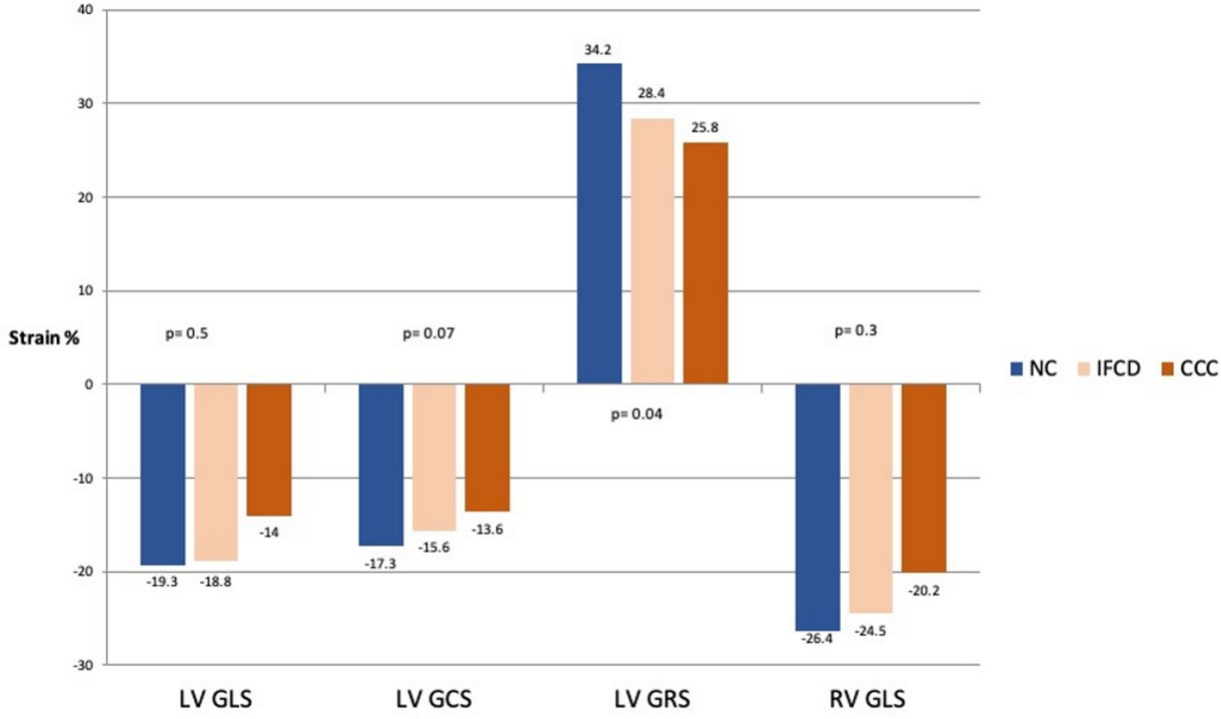
Global strain from LV (GLS, GCS, GRS) and RV (free wall) GLS in IFCD and CCC groups. Fig 2 shows median values of each studied group (NC, IFCD and CCC) of LV GLS, GCS, GRS and RV GLS. All indexes of LV and RV global deformation express a tendency of reduction from NC to IFCD and to CCC. IFCD = Indeterminate Form of Chagas disease; CCC = Chronic Chagas cardiomyopathy; LV = left ventricle; RV = right ventricle; GLS = global longitudinal strain; GCS = global circumferential strain; GRS = global radial strain. p-value of comparison between NC vs IFCD.

**Fig 3:**
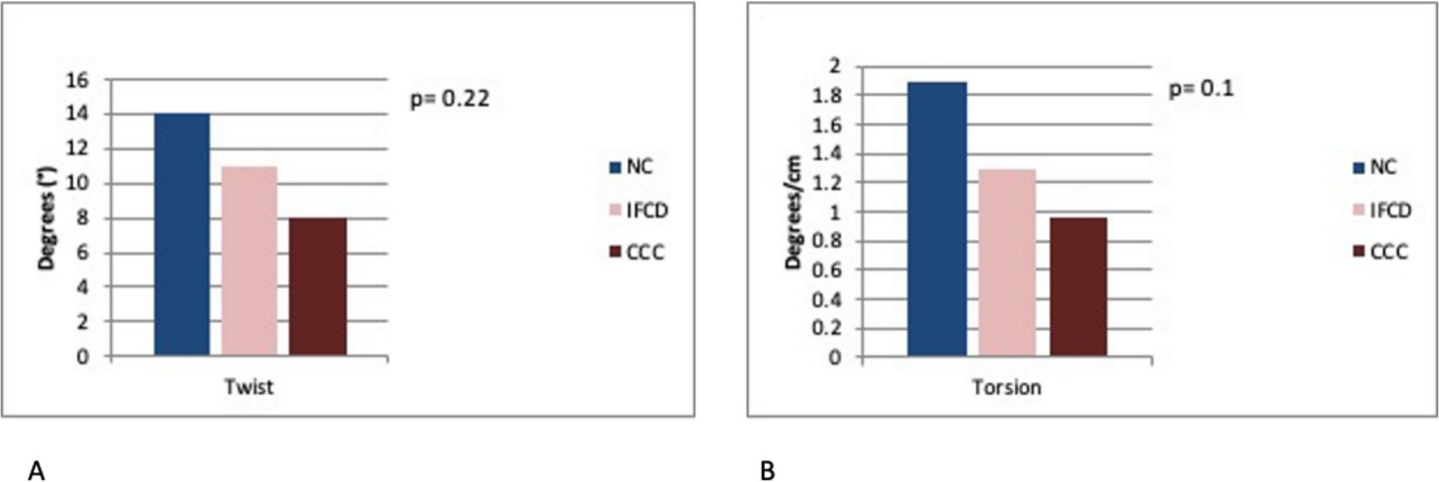
LV Twist (panel A) and torsion (panel B) between groups. Fig 3 depicts median values of twist (panel A) and torsion (panel B) with a tendency of reduction from NC to IFCD and to CCC. p-value of comparison between NC vs IFCD.

Global strain parameters GLS and GCS from both LV and RV were also similar between individuals in the IFCD and NC groups. GRS was lower in IFCD group versus the NC group (28.4 ± 14.6 vs 34.2 ± 10.3; p = 0.043) and there was a trend for being lower in the CCC group as compared to the NC group (25.8 ± 15.4 vs 34.2 ± 10.3; p = 0.058). Individuals with CCC had reduced GLS and GCS, twist and torsion in comparison with the NC group. These patients with CCC had also lower values of RV GLS from both the four-chamber view and from RV free wall. (**Table 2, Fig 2 and Fig 3)**

In comparison with the NC group, IFCD subjects had significantly reduced RegLS in LV segments 3 (basal infero-septal), 4 (basal-inferior), 9 (mid infero-septal) and 11 (mid infero-lateral)(**Table 3**). Reduction of RegLS was even more marked in patients with CCC for all segments, except for the 2 (basal antero-septal), 6 (basal antero-lateral) and 8 (mid antero-septal)(**Table 3)**.

**Table 3:** LV longitudinal regional strain comparison across the groups.

	NC	IFCD	CCC	p-value
**LV segment number**				
1. Basal Anterior	-16.7 ± 4.0	-14.6 ± 5.7	-12.2 ± 7.1^#^	0.25
2. Basal Anteroseptal	-15.5 ± 2.9	-14.8 ± 3.6	-14.1 ± 5.0	0.49
3. Basal inferoseptal	-15.2 ± 2.7	-13.1 ± 3.4*	-9.0 ± 5.9^#^	0.02
4. Basal Inferior	-18.6 ± 2.2	-16.3 ± 3.3*	-11.9 ± 5.6^#^	0.01
5. Basal Inferolateral	-16.9 ± 4.2	-15.7 ± 5.3	-7.7 ± 13.0^#^	0.46
6. Basal Anterolateral	-16.5 ± 3.5	-16.1 ± 6.4	-11.8 ± 9.2	0.84
7. Mid Anterior	-17.7 ± 3.0	-15.6 ± 4.1	-12.1 ± 7.5^#^	0.07
8. Mid Anteroseptal	-19.2 ± 3.6	-19.0 ± 3.8	-16.9 ± 6.6	0.75
9. Mid Inferoseptal	-19.4 ± 2.0	-17.7 ± 3.2*	-14.5 ± 6.3^#^	0.03
10. Mid Inferior	-19.9 ± 2.2	-18.3 ± 2.9	-13.0 ± 7.2^#^	0.05
11. Mid Inferolateral	-17.8 ± 2.8	-15.2 ± 3.5*	-10.5 ± 8.2^#^	0.01
12. Mid Anterolateral	-17.3 ± 3.2	-17.1 ± 3.9	-12.1 ± 8.3^#^	0.91
13. Apical Anterior	-21.7 ± 6.0	-21.6 ± 6.4	-12.5 ± 9.6^#^	0.96
14. Apical Septal	-22.8 ± 5.1	-23.6 ± 6.1	-13.8 ± 14.9^#^	0.65
15. Apical Inferior	-23.8 ± 4.2	-24.3 ± 5.1	-16.1 ± 12.2^#^	0.87
16. Apical Lateral	-21.4 ± 4.2	-20.7 ± 5.3	-15.1 ± 7.9^#^	0.44
17. Apex	-22.6 ± 3.8	-22.1 ± 5.6	-15.2 ± 9.4^#^	0.75

* p <0.05 NC vs IF

# p<0.05 NC vs CF

NC = Non-infected control

IFCD = Indeterminate Form Chagas Disease

CCC = Chronic Chagas Cardiomopathy

LV = left ventricle.

Myocardial fibrosis was quantified in 36 out of 45 individuals with CD (IFCD+CCC). Fibrosis was detected in 12 of them (2 individuals (8%) of IFCD, and in 10 patients (65%) of CCC group). Among CD individuals there were no significant differences in demographic characteristics between individuals with or without fibrosis. In comparison with those without fibrosis, individuals with fibrosis had significantly higher LV diameters (5.2±0.9 vs 4.5±0.5; p = 0.013) and lower left ventricle systolic function (LVEF: 45.7±13.7 vs 59.6±6.8, p = 0.001). Patients without fibrosis had significantly higher values of GLS (-18.5±3.4 vs-14.0±5.8, p = 0.006) versus those with detected fibrosis. However GCS, GRS, twist and torsion did not differ between them (**Table 4**). Fibrosis was more prevalent in the mid infero-lateral, basal infero-lateral and apex segments.

**Table 4:** Relationship of echocardiographic parameters of LV and RV ventricular mechanics and LV fibrosis in patients with Chagas disease (IFCD+CCC).

	Absence of LV Fibrosis	Presence of LV Fibrosis	p-value
n	24	12	
LV global longitudinal strain (%)	-18.5 ± 3.4	-14.0 ± 5.8	0.006
LV global circumferential strain (%)	-15.4 ± 4.1	-13.0 ± 5.3	0.159
LV global radial strain (%)	25.7 ± 15.4	23.7 ± 16.8	0.779
LV twist (degree)	9.5 ± 9.9	7.1 ± 7.5	0.498
LV torsion (degree/mm)	1.2 ± 1.3	0.8 ± 1.0	0.339
RV free wall longitudinal strain (%)	-24.4 ± 4.7	-20.8 ± 6.1	0.077
RV free wall and septum strain (%)	-18.7 ± 5.1	-17.4 ± 4.3	0.488

IFCD = Indeterminate Form CD; CCC = Chronic Chagas Cardiomyopathy; LV = Left ventricle; RV = Right ventricle.

*p<0.05

**Fig 4:**
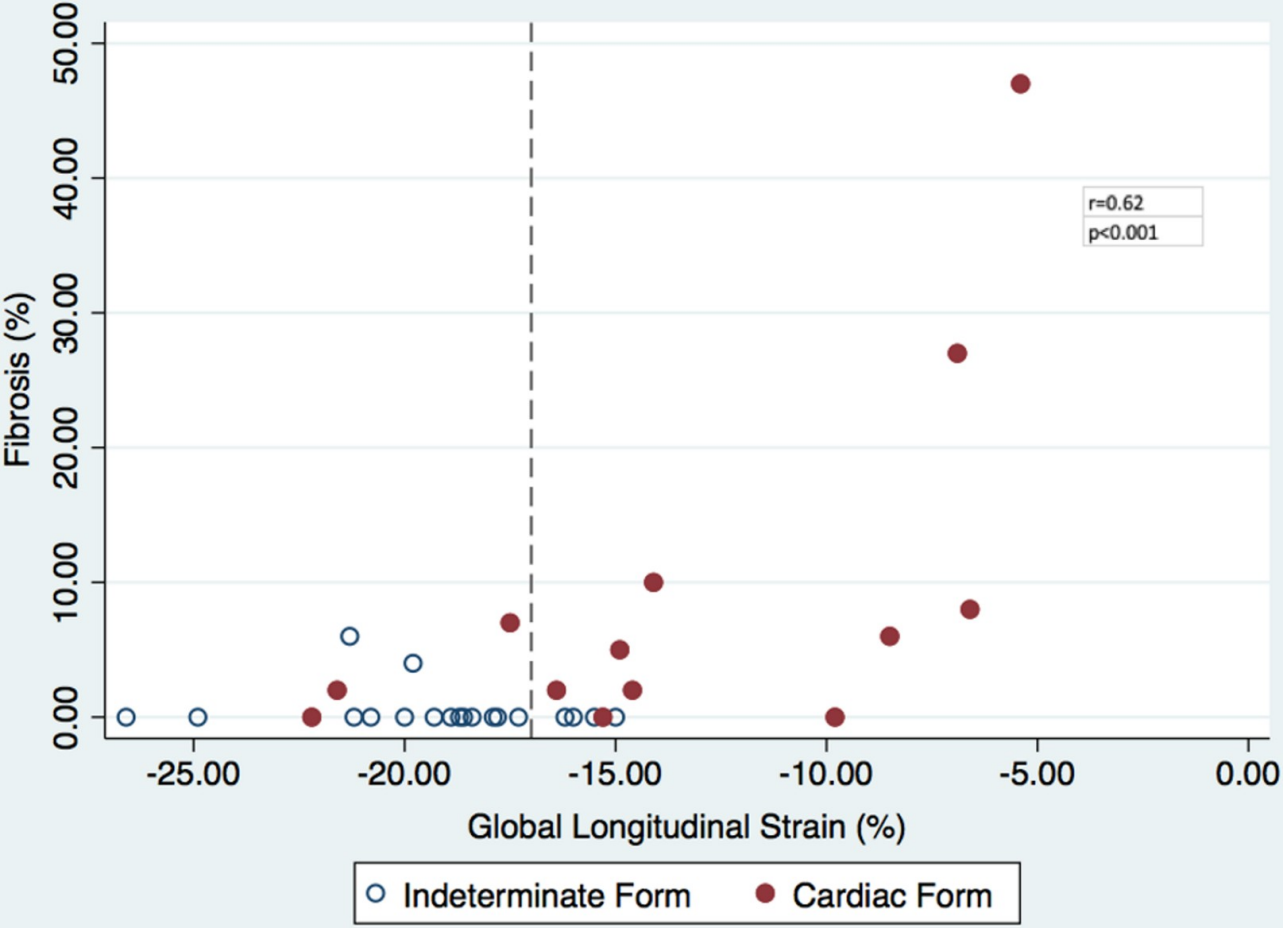
Correlation between myocardium percentage of fibrosis and GLS in patients with CD. Dotted line marks the clinical lower limit of normality of GLS for this software.

Although there was a statistically significant correlation (r = 0.625, p<0.001) between GLS and fibrosis in the whole set of patients with CD (IFCD and CCC), it was not linear (**Fig 4**). From the graphic, no significant fibrosis is detected even when GLS is below normal levels (dotted line) for this software, and only marked reductions in GLS identify patients with a significant extent of fibrosis (**Fig 4**).

**Fig 5:**
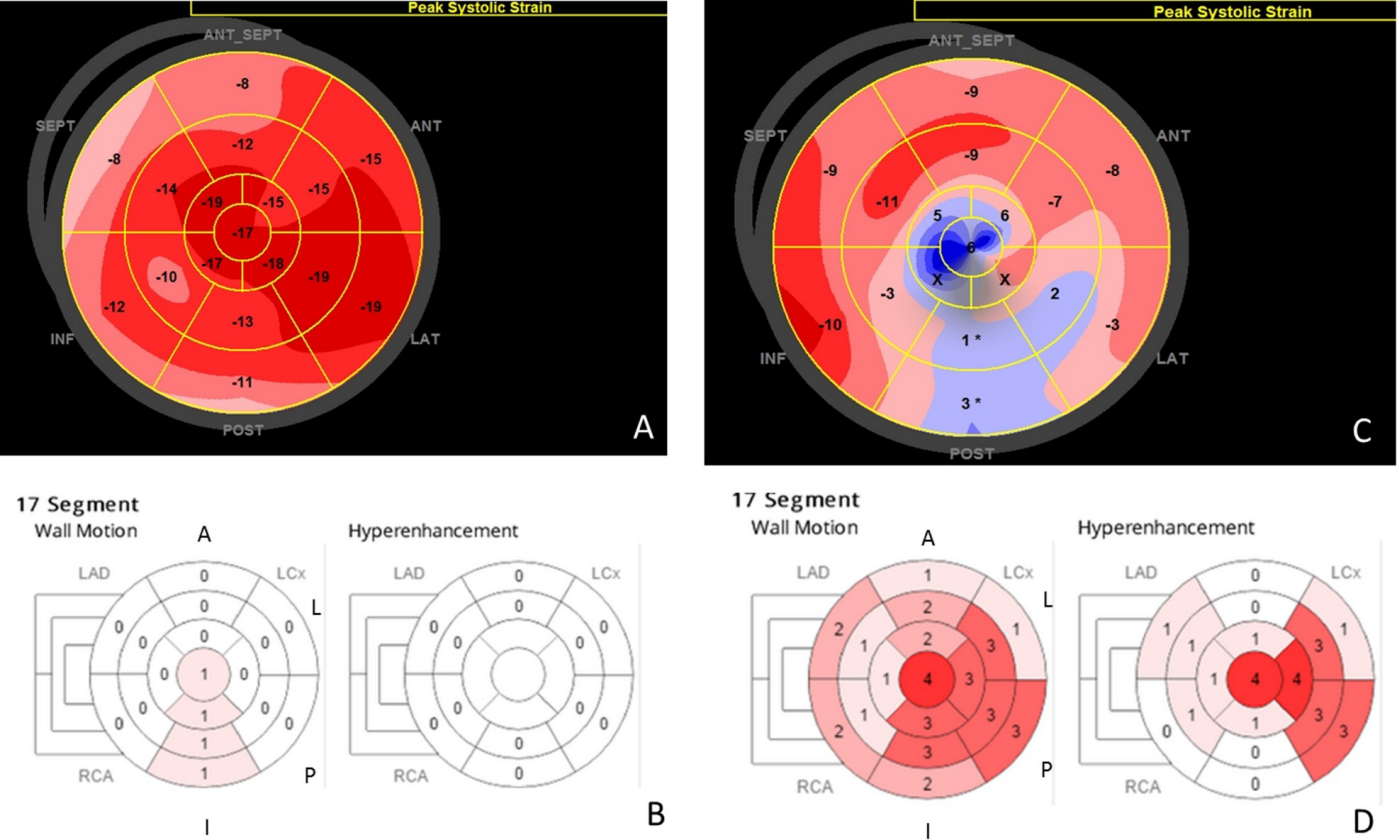
Illustrative examples of subjects with IFCD (panel A and B) and CCC (panels C and D). Panels A and C show RegLS in a bulls-eye representation. Inferior panels (B and D) represent wall motion values (left) and hyper-enhancement (fibrosis) at right. This IFCD subject (panel A and B) shows reduced RegLS even without myocardial fibrosis in LGE. CCC subject (C and D) shows worse RegLS values and there is a topographic correlation with fibrosis.

Fig 5 depicts illustrative subjects with IFCD and CCC showing how RegLS is reduced in the inferior and posterior segments of an IFCD patient even without myocardial fibrosis; this abnormality of RegLS is even more pronounced in the patient from the CCC group and it is topographically related to fibrosis in this case.

### Reproducibility

Values for intra-observer variability of GLS, GCS, GRS and twist were assessed by intra-class correlation coefficient (ICC) as follows: 0.99, 0.90, 0.86 and 0.97. ICC for inter-observer variability of GLS, GCS, GRS and twist were 0.99, 0.89, 0.86 and 0.98. Inter-observer variability of RegLS varied from 0.43 (apical lateral segment) to 0.91 (mid inferior segment).

## Discussion

The fundamental hypothesis of the study, that STE can identify early global or regional myocardial impairment in individuals at early stages of CD, was confirmed by the finding that RegLS is indeed reduced in four segments of LV (basal inferior, basal inferior-septal, mid inferior-septal and mid inferior-lateral) in the group of IFCD, despite the absence of any other evidence of LV dysfunction (LVEF, GLS or GCS), or fibrosis as detected by CMR. To the best of our knowledge this is the first investigation to show an abnormal strain pattern of early myocardial involvement in IFCD individuals who do not have detectable fibrosis. Global indexes of myocardial deformation analysis were not impaired in IFCD. However, in patients with the CCC, almost all LV segments showed markedly reduced RegLS, and the impairment of myocardial strain is correlated with the presence of regional fibrosis.

Comparison of our findings with those of previous preliminary studies using STE in the context of CD is hampered by dissimilarities in sample populations and technical approaches. For example, the findings of RegLS reduction in early stages of CD presented here are in part concordant with those reported by Barbosa et al, of reduction in RegLS in two LV segments (basal-inferior and inferior-septal), as compared to NC [24]. The concordance is seen despite the fact that the results of deformation abnormalities in their study were not correlated to any tissue characterization analysis and that strain measurement technique, if global or systolic peak, was not described. In contrast, Gomes et al measured global but not systolic peak strain, and did not find significant differences in any regional strain parameter between individuals with the IFCD and NC [25]. However, when evaluating a small subgroup of 7 IFCD individuals with fibrosis detected by CMR they found reduced RegLS in the basal inferior-septal segment although this abnormality was associated with reductions of GLS, GCS and GRS in this subgroup. Even considering that their IFCD sample comprised a small group (4.8%) of patients with left anterior fascicular block, who could not be characterized as having the IFCD by stringent criteria of normal EKG, there were no differences in RegLS between groups. In fact, conduction defects change regional patterns of strain [33] and the choice of authors to measure global peak instead of systolic peak could explain such differences from our data. When measuring systolic peak, as recommended by strain taskforces [31, 34], regional differences were apparent in our study.

It is important to emphasize that the results of our study, reinforcing previous data which showed that LV global myocardium deformation strain indexes GLS and GCS are still preserved in presumably early phases of CD [22, 24, 25], are indirectly corroborated by a more recent cross-sectional study in patients with different Chagas cardiomyopathy stages by Echeverría et al. They also reported that a GLS value with a cut-off of -20.5% could detect the onset of myocardial alterations, despite the fact that this is not a low value of normality in most dedicated software [35].

STE measurements in our study were strictly controlled. This is the first study using STE in patients with CD which strictly followed the recently published standardization document to measure myocardial strain [34]. Strain values were measured as end-systolic (ESS) peak and timing of ESS was defined as time of aortic valve closure by Doppler signal when measuring LV parameters and as time to pulmonic valve closure when measuring RV parameters. In contrast to our study, definitions of timing of measuring peak strain were not always consistent in previous reports on CD [22, 24, 25]. We postulate that, considering the high prevalence of conduction disturbances in CD patients [36], exact systolic temporal definitions may have a major impact on strain measurements, so that this factor may have contributed to explain some of the discrepant findings among the various studies [37].

Measurement of GRS has yielded more controversial data in studies of patients with diverse forms of CD [22]. Results of our study may be due to the large standard deviation of this parameter, and to the various technical factors that may influence GRS values. Although STE technique is considered angle independent it is still dependent upon image quality. Measurements of speckles can be made in any direction, but speckle size is not uniform in every region of bidimensional echocardiographic images because of lateral resolution. When imaging LV from the apical window to measure longitudinal deformation, the effect of lateral resolution may not have the same importance as it has in lateral and septal segments of short axis views. The difficulty in assessing speckles into lateral sides of sector image is increased by the fact that radial strain is measured as a displacement of speckles in relation to a central reference point in LV cavity. All of these factors can play an important role in interpreting variability of radial strain data as discussed above.

This study also shows that LV twist and torsion values are reduced only in advanced forms of CD, while previous data reported in a small subset of 9 patients with IFCD by García-Álvarez et al found reduction in twist values compared to controls [22]. In contrast, our results are in agreement with those of Lima et al, who showed no difference in twist or torsion in IFCD as compared to NC [23]. However, these authors did not find abnormal values of twist or torsion in their CCC group of patients, possibly explained because of age differences in groups. Considering twist as an expression of circular deformation of both LV base and apex, this parameter is probably more dependent upon the performance of circumferential myocardial fibers, which are predominantly located in myocardial mid-wall [28, 32]. Since the mid-wall is the primary location of fibrosis in CD [38], it is therefore understandable that twist and torsion can be abnormal in patients with CCC, as detected in our study, but not in IFCD, when GCS is still preserved.

Myocardial fibrosis detected by CMR has been shown to have prognostic value in patients with CCC [39, 40]. However, our results suggest that regional myocardial deformation impairment in early stages of CD appear before myocardial fibrosis. Other mechanisms of myocardial damage such as edema or inflammation, could be responsible for this, similarly what has been reported in other cardiomyopathies [37, 41]. Studies with the hamster experimental model of CCC demonstrated early regional LV systolic dysfunction correlated to inflammation but not of myocardial fibrosis [42]. It is noteworthy that whatever the underlying mechanisms responsible for it may be, regional myocardial dysfunction, as expressed by the wall motion segmental score index (WMSi), was significantly and independently related to death in 1,508 CCC patients followed for 5.4 years in the BENEFIT trial [30].

An important consideration about our results is that fewer individuals (8%) with IFCD had myocardial fibrosis in comparison with previously reported data from other IFCD series (12%-27% of subjects) [43]. Several reasons may be responsible for these discrepancies. Different from other reported series, in which patients were enrolled from cardiologic clinics or specialized tertiary centers [24, 44], in our study subjects were selected from a broad population (n = 325) of serologically positive but asymptomatic subjects, which were not patients of any cardiologic clinic. Moreover, they were subjects tested serologically for CD because of less ominous conditions, such as a pre-operatory evaluation, blood donation, or other non-cardiologic situation. Furthermore, it is relevant to emphasize that in some studies using STE the IFCD was not fully characterized according to the classical definition [24, 25]. These contrasts with our more stringent methodology for selection of subjects with IFCD; the clinical criteria in selecting IFCD patients were strictly fulfilled, such as a completely normal EKG and chest X-ray.

### Clinical implications

Recognizing an abnormal regional strain pattern in CD can guide the detection of cardiac involvement even in early stages of the disease. It can be helpful to identify this entity in the group of patients with suspected non-ischemic cardiomyopathies who still present a diagnostic challenge [45], mainly in non-endemic countries, where serological testing is not used as screening.

Early detection of regional myocardial damage can help design strategies for follow-up and management of this group of CD patients. Since regional myocardial impairment has been prognostic in this population, treatment strategies could be driven, in the future, by assessment of RegLS.

### Limitations

We recognize sample size is a limitation. However, we based sample size on the underlying hypothesis of the study and followed prospectively devised strict criteria to screen 325 subjects and to select a group of 25 individuals of IFCD.

There was a significant but random loss of CMR data for analysis of fibrosis. Although all CD subjects were prospectively studied with CMR at the same day of echocardiography, which is an important and costly aspect of the study, still a proportion of 38% in the CCC group and 23.3% in the IFCD group could not undergo fibrosis assessment because of technical problems with acquired images. Also, this study did not provide evaluation of oedema and inflammation by CMR images to test the hypothesis of early impairment of strain indexes being related to those pathological changes, rather than to fibrosis.

Finally, the cross-sectional design of the study does not allow conclusions about the prognostic importance of the abnormal RegLS pattern in early stages of CD. Further studies with specific design should be performed to address this question. The results of an on-going such study should soon become available [46].

## Conclusions

RegLS is reduced in the infero-septal, inferior and inferior-lateral LV segments in subjects with IFCD in absence of impairment of global indexes of myocardial deformation and of detectable myocardial fibrosis. Patients with CCC presented a markedly abnormal strain pattern, associated to reduction of LV GLS, GCS, twist and torsion and also of RV GLS.

### Perspectives

Chronic cardiomyopathy caused by Chagas disease is the most prevalent of nonischemic cardiomyopathies in endemic countries of Latin America and because of migration movements during the last decades its prevalence is augmenting also in North America and Europe. RegLS is reduced in subjects with the IFCD even before myocardium fibrosis or any other signs of cardiac involvement. In the CCC group of patients there is impairment of global strain indexes from LV as GLS and GCS, twist and torsion and also RV GLS.
